# Pro-Oxidative Effect of KIO_3_ and Protective Effect of Melatonin in the Thyroid—Comparison to Other Tissues

**DOI:** 10.3390/life11060592

**Published:** 2021-06-21

**Authors:** Paulina Iwan, Jan Stepniak, Malgorzata Karbownik-Lewinska

**Affiliations:** 1 Department of Oncological Endocrinology, Medical University of Lodz, 7/9 Zeligowski St., 90-752 Lodz, Poland; paulina.iwan@op.pl (P.I.); jan.stepniak@umed.lodz.pl (J.S.); 2 Polish Mother’s Memorial Hospital—Research Institute, 281/289 Rzgowska St., 93-338 Lodz, Poland

**Keywords:** melatonin, potassium iodate, KIO_3_, lipid peroxidation, antioxidant, salt iodization, thyroid

## Abstract

Not only iodine deficiency, but also its excess may contribute to thyroid cancer. Potassium iodate (KIO_3_), which is broadly used in the salt iodization program, can increase oxidative damage to membrane lipids (lipid peroxidation, LPO) under experimental conditions, with the strongest damaging effect at KIO_3_ concentration of ~10 mM (corresponding to physiological iodine concentration in the thyroid). Melatonin is an effective antioxidant, which protects against KIO_3_-induced LPO in the thyroid. This study aimed to compare the protective effects of melatonin, used in the highest achievable in vitro concentration, against KIO_3_-induced oxidative damage to membrane lipids in various porcine tissues (thyroid, ovary, liver, kidney, brain, spleen, and small intestine). Homogenates were incubated in the presence of KIO_3_ (20; 15; 10; 7.5; 5.0; 0.0 mM) without/with melatonin (5 mM). The malondialdehyde + 4-hydroxyalkenals (MDA + 4-HDA) concentration (LPO index) was measured spectrophotometrically. KIO_3_ increased the LPO in all examined tissues; in the thyroid, the damaging effect of KIO_3_ (10; and 7.5 mM) was lower than in other tissues and was not observed for the lowest concentration of 5 mM. Melatonin reduced LPO induced by KIO_3_ (10, 7.5, and 5 mM) in all tissues, and in the thyroid it was also protective against as high a concentration of KIO_3_ as 15 mM; the LPO level resulting from KIO_3_ + melatonin treatment was lower in the thyroid than in other tissues. In conclusion, the thyroid is less sensitive tothe pro-oxidative effects of KIO_3_ compared to other tissues. The strongest protective effect of melatonin was observed in the thyroid, but beneficial effects were significant also in other tissues. Melatonin should be considered to avoid the potential damaging effects of iodine compounds applied in iodine prophylaxis.

## 1. Introduction

Free radicals and reactive oxygen species (ROS) are highly reactive transient molecules produced by almost all aerobic cells [1]. ROS include both oxygen radicals (e.g., superoxide anion radical—O_2_^•−^, hydroxyl radical—^•^OH, and hydroperoxyl radical—^•^OOH) and certain nonradical oxidizing agents easily converted into radicals (e.g., ozone—O_3_, hydrogen peroxide—H_2_O_2_, hypochlorous acid—HOCl) [2,3]. There is a balance between production and detoxification of ROS under physiological conditions in living organisms [1]. Any imbalance between these processes may result in oxidative stress which, in turn, may cause oxidative damage to membrane lipids, DNA and proteins [3,4]. The importance of oxidative stress is commonly emphasized in the pathogenesis of various degenerative diseases, such as cardiovascular and neurodegenerative diseases, kidney diseases, diabetes or cancer [5].

Due to the high level of polyunsaturated fatty acids (PUFAs) in cellular and organelle membranes, they are especially susceptible to lipid peroxidation (LPO), a process in which free radicals remove electrons from lipids and subsequently produce reactive intermediates. LPO damages phospholipids directly—it can act as a cell death signal—and it is implicated in various degenerative processes, including cancer [4,6].

Although oxidative reactions occur in almost all tissues and organs, the thyroid gland is the organ of “oxidative nature” [7]. ROS are essential for thyroxine (T_4_) synthesis since, H_2_O_2_ produced in thyroid follicular cells is indispensable in attaching iodine atoms to thyroglobulin [8]. Therefore, the thyroid gland is characterized by a high level of oxidative stress, which—in response to additional oxidative abuse caused by various prooxidants—may lead to different thyroid diseases, such as thyroid cancer [7]. Additionally, excess of iodine, as an exogenous pro-oxidant, may induce apoptosis in thyroid follicular cells [9].

An important role in iodine homeostasis is played by the sodium/iodide symporter (NIS). This protein is responsible for active transport of iodide (I^−^) into the thyroid gland at the level of the basolateral membrane [10]. NIS was documented to mediate I^-^ transport not only in the thyroid gland, but also in other tissues, which are able to concentrate radioiodine, such as lactating breast, salivary glands, stomach, and small intestine [11,12]. Thyrotropin (TSH) is the primary regulator of I^−^ uptake and NIS expression, but only in thyroid follicular cells [11]. It is worth mentioning that NIS mRNA has also been found in other tissues, such as colon, ovaries, uterus or spleen, but the role and significance of NIS in these tissues is still unclear [10].

Iodine is a micronutrient playing an essential role in metabolism. Its deficiency may lead to goiter and hypothyroidism and in pregnant patients to impaired infant neurobehavioral development [13,14,15]. Correction of iodine deficiency may decrease the prevalence of goiter and shift thyroid cancer subtypes towards a less malignant form and ensure adequate thyroid hormone synthesis [15]. However, not only iodine deficiency, but also its excess may cause pathological phenomena such as thyroiditis, hypo- or hyperthyroidism, and papillary thyroid cancer [13].

Universal salt iodization is widely recognized as the most cost-effective method to reduce iodine deficiency [16]. Programs of salt iodization are based on the use of either potassium iodide (KI) or potassium iodate (KIO_3_), with the latter—due to its higher stability—being the most commonly used iodine compound for this process [16]. Both KI and KIO_3_ have different pro- and antioxidative properties; KI is the reductant, whereas KIO_3_ is the oxidant and may react with oxidizable substances [17]. The differences between the oxidative properties of KI and KIO_3_ and their effects on oxidative damage to macromolecules in the thyroid gland were documented recently [18,19,20]. KI, used in the doses recommended in iodine prophylaxis, may prevent oxidative damage to membrane lipids in the thyroid [18]. In turn, KIO_3_ damages membrane lipids in the thyroid with the strongest damaging effect observed at concentrations of around 10 mM [18] and 15 mM [20,21,22], which correspond to the physiological iodine concentration in the thyroid [23,24,25].

The total body iodine content in humans was estimated to be 12–25 mg, of which 5–15 mg is stored in the thyroid [26], although data concerning this issue do vary. In another study using pigs, the distribution of iodine in the organism was similar, i.e., the thyroid contained about 80% of the total body iodine, internal organs and blood (14%), muscle and fat (5%), and bones (1%) [27]. Compared to the thyroid gland, the extrathyroidal tissues contain only traces of iodine. The ratio of the iodine concentration in kidney, liver, muscle and skin to that in the thyroid gland was calculated as 1 to 100,000 [28]. However, even in tissues with a low level of iodine concentrations such as the gastrointestinal tract, kidneys or liver, high doses of KIO_3_ have shown potential toxicity [29].

Melatonin, N-acetyl-5-methoxytryptamine, being a tryptophan metabolite, mainly produced by the pineal gland, is very strong and effective in reducing oxidative stress [30]. It is considered that melatonin exists possibly in all animal and plant species. Probably melatonin appeared 3.0–2.5 billion years ago in photosynthetic cyanobacteria as an antioxidant [31]. It is documented that melatonin reveals protective effects against oxidative stress not only in the thyroid gland [20,22], but also, as was even earlier found, in many other tissues and organs, among others in kidney [32], spleen [33], ovary [34], liver [35] or erythrocytes [36].

Although the antioxidant capacity of melatonin has been proven both in vitro and in vivo conditions, there are few studies in which melatonin revealed pro-oxidative properties. It has been found, for example, that melatonin promotes the generation of ROS when used in a certain range of concentrations (mainly from μM to mM) and, additionally, depending on duration of the treatment under in vitro conditions [37]. What is of great importance is that this pro-oxidative action of melatonin was observed mostly in cancer cells and promoted inflammatory responses and apoptosis [37]. This observation has not been confirmed until now under in vivo conditions, but the ability of melatonin to induce apoptosis in tumor cells might have important therapeutic implications [37].

In our previous studies [20,22] we observed, that melatonin was able to reduce oxidative damage to membrane lipids caused by KIO_3_, when this prooxidant was used in doses close to physiological concentrations of iodine in the thyroid. In the present study we decided to compare the protective effects of melatonin against KIO_3_-induced oxidative damage to membrane lipids in various porcine tissues, i.e., in the thyroid, the ovary, the liver, the kidney, the brain, the spleen, and the small intestine. KIO_3_ was used in the range of concentrations comprising those corresponding to physiological iodine concentration in the thyroid, whereas melatonin was used in the highest achievable in vitro concentration (i.e., 5 mM).

## 2. Materials and Methods

### 2.1. Chemicals

Potassium iodate (KIO_3_) and melatonin were purchased from Sigma (St. Louis, MO, USA). The ALDetect Lipid Peroxidation Assay Kit was obtained from Enzo Life Sciences, Inc. (Zandhoven, Belgium). All used chemicals were of analytical grade and came from commercial sources.

### 2.2. Animals

Porcine tissues (i.e., thyroid, ovary, spleen, liver, brain, small intestine, and kidney) were collected from fifteen (15) female animals at a slaughter-house, frozen on solid CO_2_ and stored at −80 °C until assayed. Each experiment was repeated three times.

### 2.3. Incubation of Tissue Homogenates

Porcine tissues (thyroid, ovary, spleen, liver, brain, small intestine, and kidney) were homogenized in ice cold 20 mM Tris-HCl buffer (pH 7.4) (10%, *w*/*v*) and then incubated for 30 min at 37 °C in the presence of KIO_3_ (20; 15; 10; 7.5; 5.0, 0.0 mM) without or with addition of melatonin in a concentration of 5 mM (the highest achievable concentration resulting from its limited solubility).

The concentrations of KIO_3_ and melatonin were chosen on the basis of the results of our previous studies [18,19,20,22].

The reactions were stopped by cooling the samples on ice.

### 2.4. Measurement of Lipid Peroxidation Products

The concentrations of malondialdehyde + 4-hydroxyalkenals (MDA + 4-HDA), as an index of lipid peroxidation, were measured in homogenates with the ALDetect Lipid Peroxidation Assay Kit. The homogenates were centrifuged at 5000× *g* for 10 min at 4 °C. After obtaining supernatant, each experiment was carried out in duplicate. The supernatant (200 μL) was mixed with 650 μL of a methanol:acetonitrile (1:3, *v*/*v*) solution, containing a chromogenic reagent, N-methyl-2-phenylindole, and vortexed. Following the addition of 150 μL of methanesulfonic acid (15.4 M), the incubation was carried out at 45 °C for 40 min. The reaction between MDA + 4-HDA and N-methyl-2-phenylindole yields a chromophore, which is spectrophotometrically measurable at an absorbance of 586 nm, using a solution of 10 mM 4-hydroxynonenal as the standard. The level of lipid peroxidation is expressed as the amount of MDA + 4-HDA (nmol) per mg protein. Protein was measured using Bradford’s method, with bovine albumin as the standard [38].

### 2.5. Statistical Analyses

The data were statistically analyzed, using a one-way analysis of variance (ANOVA), followed by the Student–Neuman–Keuls’ test, or using an unpaired t-test. Statistical significance was determined at the level of *p* < 0.05. Results are presented as means ± SE.

## 3. Results

**Figure 1:**
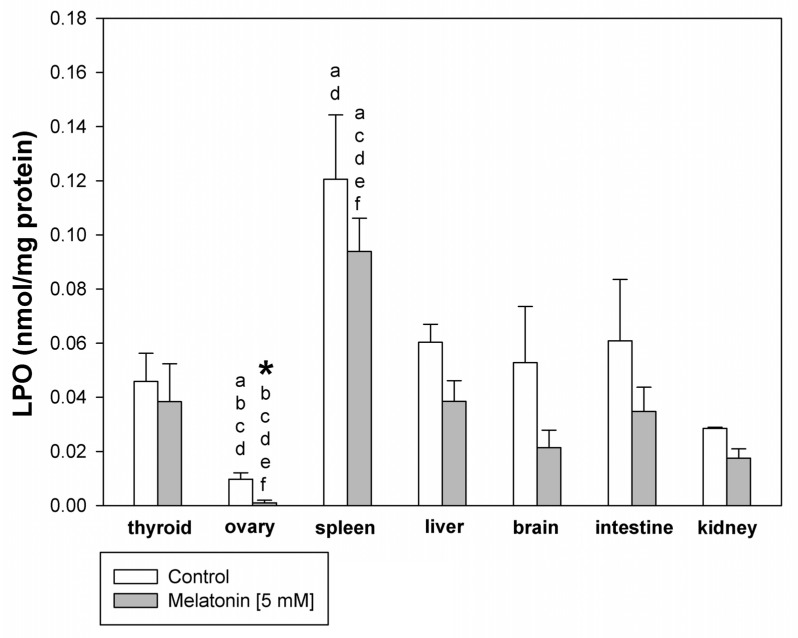
Lipid peroxidation, measured as MDA + 4-HDA level, in homogenates of porcine tissues (thyroid, ovary, spleen, liver, brain, small intestine, kidney), incubated without any substance (control; white bars), or with melatonin [5 mM] (grey bars). *—*p* < 0.05 vs. control (without melatonin) in the same tissue; **a**—*p* < 0.05 vs. respective bar (control or melatonin) in the thyroid; **b**—*p* < 0.05 vs. respective bar (control or melatonin) in the spleen; **c**—*p* < 0.05 vs. respective bar (control or melatonin) in the liver; **d**—*p* < 0.05 vs. respective bar (control or melatonin) in the kidney; **e**—*p* < 0.05 vs. respective bar (control or melatonin) in the brain; **f**—*p* < 0.05 vs. respective bar (control or melatonin) in the intestine.

The basal level of LPO was lower in the ovary than in all other tissues, which was statistically confirmed for the thyroid, spleen, liver, and kidney. In turn, the basal level was higher in the spleen than in other tissues, which was significant and confirmed for thyroid, ovary, and kidney. The incubation with melatonin decreased the basal level of LPO only in ovary tissue (Figure 1).

**Figure 2:**
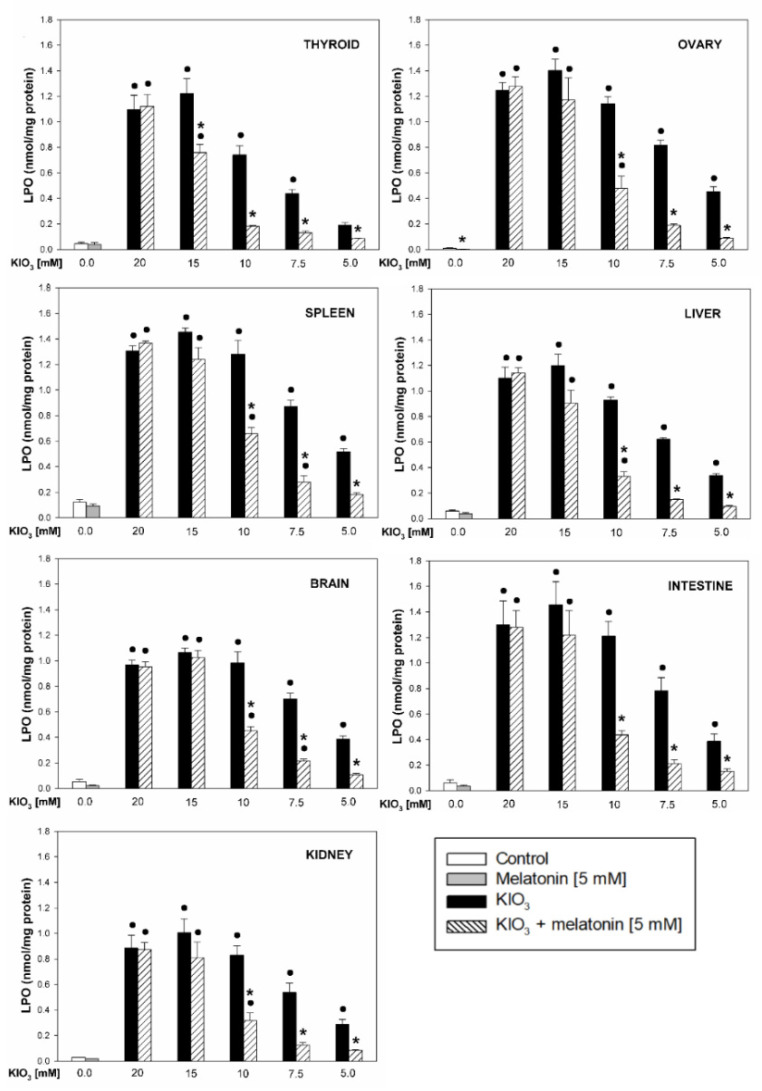
Lipid peroxidation, measured as MDA + 4-HDA level, in homogenates of porcine tissues (thyroid, ovary, spleen, liver, brain, small intestine, kidney), incubated without any substance (control; white bars), or with melatonin (5 mM) (grey bars), or with KIO_3_ (20; 15; 10; 7.5; 5.0 mM) (black bars), or with KIO_3_ (20; 15; 10; 7.5; 5.0 mM) + melatonin (5 mM) (striped bars). *●*—*p* < 0.05 vs. respective control (either without any substance or with melatonin); *—*p* < 0.05 vs. KIO_3_ in the same concentration.

**Figure 3:**
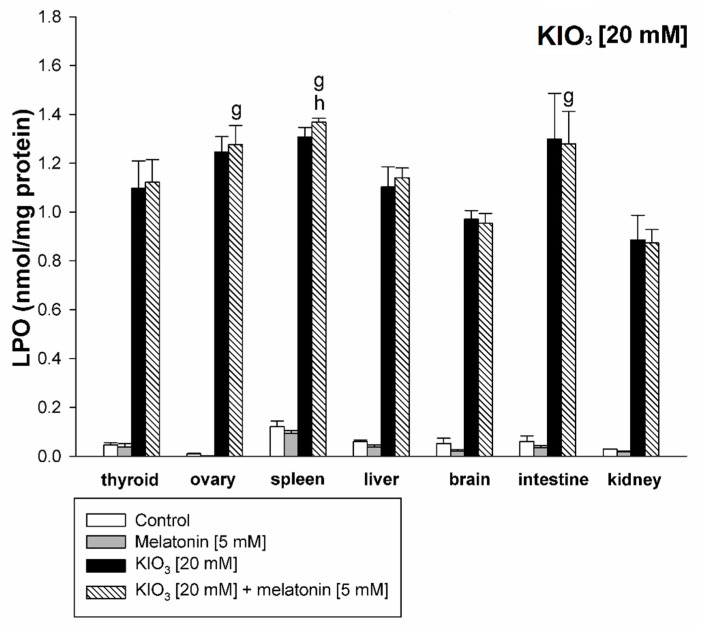
Lipid peroxidation, measured as MDA + 4-HDA level, in homogenates of porcine tissues (thyroid, ovary, spleen, liver, brain, small intestine, kidney), incubated without any substance (control; white bars), or with melatonin (5 mM) (grey bars), or with KIO_3_ (20 mM) (black bars), or with KIO_3_ (20 mM) + melatonin (5 mM) (striped bars). **g**—*p* < 0.05 vs. KIO_3_ (20 mM) + melatonin (5 mM) in kidney; **h**—*p* < 0.05 vs. KIO_3_ (20 mM) + melatonin (5 mM) in brain.

**Figure 4:**
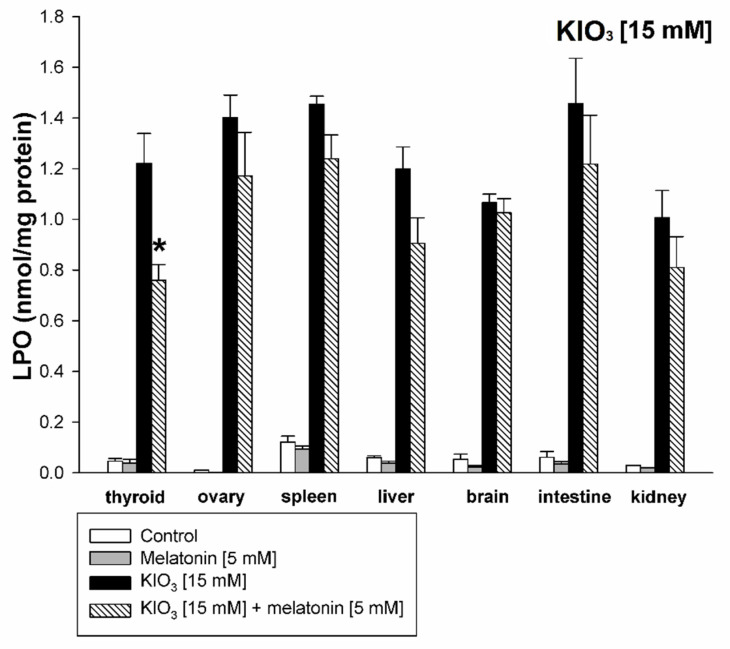
Lipid peroxidation, measured as MDA + 4-HDA level, in homogenates of porcine tissues (thyroid, ovary, spleen, liver, brain, small intestine, kidney), incubated without any substance (control; white bars), or with melatonin (5 mM) (grey bars), or with KIO_3_ [15 mM] (black bars), or with KIO_3_ (15 mM) + melatonin (5 mM) (striped bars). *—*p* < 0.05 vs. KIO_3_ in the same tissue.

**Figure 5:**
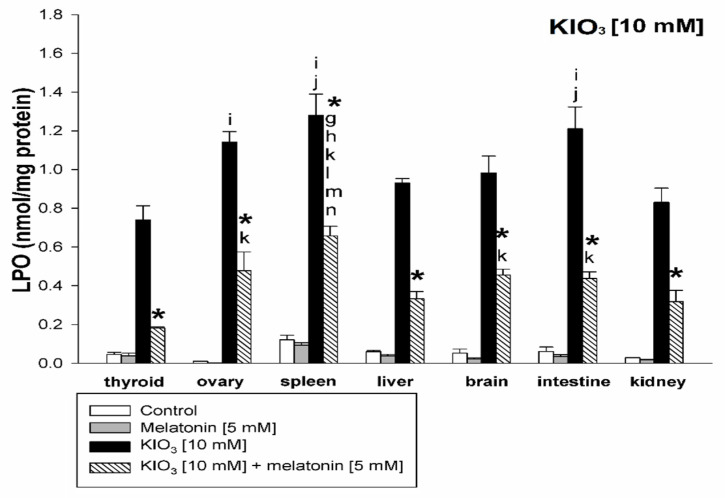
Lipid peroxidation, measured as MDA + 4-HDA level, in homogenates of porcine tissues (thyroid, ovary, spleen, liver, brain, small intestine, kidney), incubated without any substance (control; white bars), or with melatonin [5 mM] (grey bars), or with KIO_3_ [10 mM] (black bars), or with KIO_3_ [10 mM] + melatonin [5 mM] (striped bars). *—*p* < 0.05 vs. KIO_3_ in the same tissue; **i**—*p* < 0.05 vs. KIO_3_ [10 mM] in thyroid; **j**—*p* < 0.05 vs. KIO_3_ [10 mM] in kidney; **g**—*p* < 0.05 vs. KIO_3_ [10 mM] + melatonin [5 mM] in kidney; **h**—*p* < 0.05 vs. KIO_3_ [10 mM] + melatonin [5 mM] in brain; **k**—*p* < 0.05 vs. KIO_3_ [10 mM] + melatonin [5 mM] in thyroid; **l**—*p* < 0.05 vs. KIO_3_ [10 mM] + melatonin [5 mM] in ovary; **m**—*p* < 0.05 vs. KIO_3_ [10 mM] + melatonin [5 mM] in liver; **n**—*p* < 0.05 vs. KIO_3_ [10 mM] + melatonin [5 mM] in intestine.

**Figure 6:**
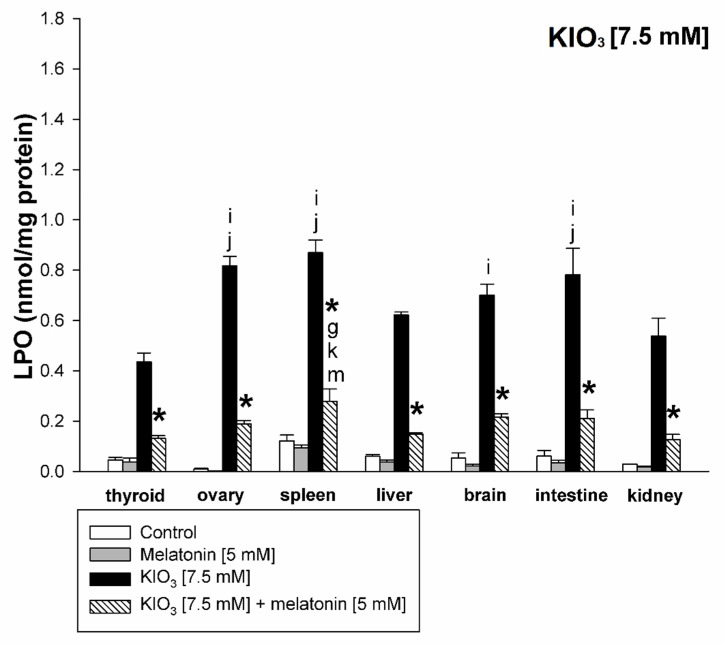
Lipid peroxidation, measured as MDA + 4-HDA level, in homogenates of porcine tissues (thyroid, ovary, spleen, liver, brain, small intestine, kidney), incubated without any substance (control; white bars), or with melatonin [5 mM] (grey bars), or with KIO_3_ [7.5 mM] (black bars), or with KIO_3_ [7.5 mM] + melatonin [5 mM] (striped bars). *—*p* < 0.05 vs. KIO_3_ in the same tissue; **i**—*p* < 0.05 vs. KIO_3_ [10 mM] in thyroid; **j**—*p* < 0.05 vs. KIO_3_ [10 mM] in kidney; g—*p* < 0.05 vs. KIO_3_ [10 mM] + melatonin [5 mM] in kidney; **k**—*p* < 0.05 vs. KIO_3_ [10 mM] + melatonin [5 mM] in thyroid; **m**—*p* < 0.05 vs. KIO_3_ [10 mM] + melatonin [5 mM] in liver.

**Figure 7:**
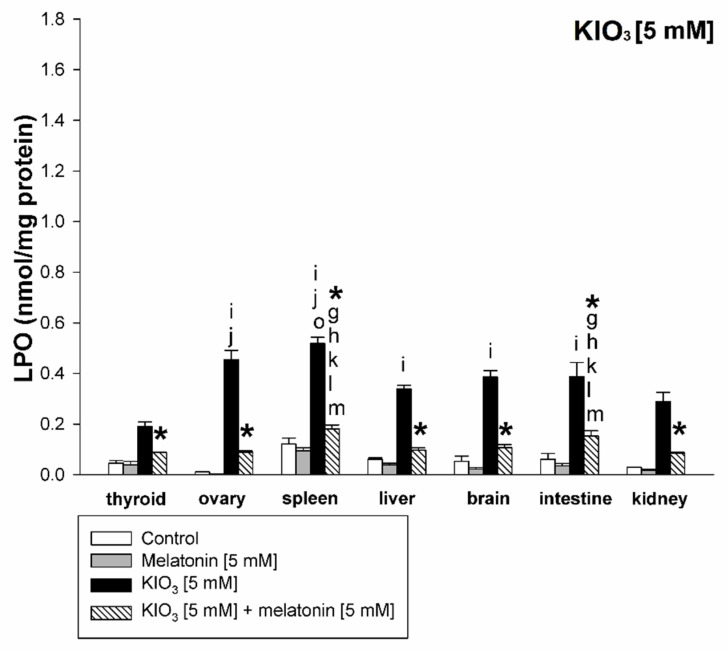
Lipid peroxidation, measured as MDA + 4-HDA level, in homogenates of porcine tissues (thyroid, ovary, spleen, liver, brain, small intestine, kidney), incubated without any substance (control; white bars), or with melatonin [5 mM] (grey bars), or with KIO_3_ [5 mM] (black bars), or with KIO_3_ [5 mM] + melatonin [5 mM] (striped bars). *—*p* < 0.05 vs. KIO_3_ in the same tissue; i—*p* < 0.05 vs. KIO_3_ [10 mM] in thyroid; **j**—*p* < 0.05 vs. KIO_3_ [10 mM] in kidney; o—*p* < 0.05 vs. KIO_3_ [10 mM] in liver; **g**—*p* < 0.05 vs. KIO_3_ [10 mM] + melatonin [5 mM] in kidney; **h**—*p* < 0.05 vs. KIO_3_ [10 mM] + melatonin [5 mM] in brain; **k**—*p* < 0.05 vs. KIO_3_ [10 mM] + melatonin [5 mM] in thyroid; **l**—*p* < 0.05 vs. KIO_3_ [10 mM] + melatonin [5 mM] in ovary; **m**—*p* < 0.05 vs. KIO_3_ [10 mM] + melatonin [5 mM] in liver.

KIO_3_ increased the lipid peroxidation in all examined tissues (i.e., thyroid, ovary, spleen, liver, brain, small intestine, and kidney) with the strongest damaging effect observed at concentrations of of 20 mM (Figure 2 and Figure 3), of 15 mM (Figure 2 and Figure 4), and of 10 mM (Figure 2 and Figure 5) vs. 7.5 mM and 5.0 mM in all tissues, and at concentrations of 20 mM (Figure 2 and Figure 3), of 15 mM (Figure 2 and Figure 4) vs. 10 mM in the thyroid and the liver. It should be stressed, however, that in thyroid tissue the damaging effect of KIO_3_ was not observed at its lowest concentration of 5 mM (Figure 2). Additionally, LPO induced by KIO_3_ at concentrations of 10 mM and 7.5 mM was significantly lower in the thyroid than in other examined tissues except the kidney (Figure 5, Figure 6 and Figure 7).

Melatonin (5 mM) reduced KIO_3_-induced lipid peroxidation in all examined tissues when this pro-oxidant was used at concentrations of 10 mM, 7.5 mM and 5 mM (Figure 2). An important observation is that in the thyroid gland, melatonin revealed a protective effect also against a higher concentration of KIO_3_, i.e., 15 mM (Figure 2 and Figure 4). The LPO level resulting from KIO_3_ + melatonin treatment was lower in the thyroid than in other tissues (Figure 5, Figure 6 and Figure 7). The latter two observations suggest that the protective effect of melatonin was the strongest in the thyroid.

## 4. Discussion

This study is the next in line, in which we evaluated antioxidative properties of melatonin against oxidative damage caused by KIO_3_, and presumably the first attempt to compare the protective effects of melatonin in various porcine tissues. For the present study we chose the concentrations of KIO_3_ (i.e., 20; 15; 10; 7.5; 5.0 mM) which had revealed the strongest damaging effect to membrane lipids in thyroid homogenates in our previous studies [18,19,20,22]. Due to the similarity between human and porcine thyroid (hormone synthesis, volume) [39] we decided to continue our experimental model also using other porcine tissues.

Although concentrations of iodine in all other tissues are much lower than in the thyroid gland [28], damaging effects of KIO_3_ were observed in all tissues examined in our study.

When we compared the damaging effect of KIO_3_ we observed that LPO induced by this compound was significantly lower in the thyroid gland than in any other examined porcine tissues (except kidney). This observation illustrates the fact that the thyroid gland has adapted to maintain large concentrations of iodine. As the thyroid constitutes an organ, in which oxidative processes are indispensable for proper functioning and thyroid hormone synthesis, some protective mechanisms have been developed to protect this gland against the huge amount of iodine. One of the thyroidal adaptations to iodine excess is the Wolff–Chaikoff effect. This effect, still not completely explained, was observed in rats exposed to high amounts of iodide, which resulted in transient reduction in the thyroid hormone synthesis; the block lasted approx. 24 h [40]. This adaptation is associated with a decrease in expression of the sodium-iodide symporter (NIS), resulting in reduced intrathyroidal iodine concentration; thus, this is the next mechanism contributing in maintaining proper thyroid function. NIS is an intrinsic membrane protein, found mainly in the basolateral membrane of thyroid follicular cells; its regulator is not only TSH, but also I^-^ itself [40,41,42,43].

The basal level of LPO was lower in the ovary than in the thyroid homogenates, which was confirmed also in our previous studies [44]; and observations from two different studies [45,46]. On the other hand, LPO induced by KIO_3_, similar to LPO induced by Fenton reaction substrates [44], was higher in the ovary than in the thyroid homogenates. This observation also confirms the hypothesis, that in physiological conditions oxidative stress in the thyroid (resulting mostly from oxidative reactions indispensable for thyroid hormone synthesis) is at a substantially higher level than in other tissues. At the same time this physiologically high level of oxidative stress in the thyroid makes this organ less vulnerable to pro-oxidative agents, such as iodate or iron (used in the Fenton reaction).

We also observed a significantly lower LPO level induced by KIO_3_ in the kidney compared to other tissues (Figure 5, Figure 6 and Figure 7). This observation may be justified by the following reason. Potassium bromate (KBrO_3_)—halogenate salt, belonging to the same class (oxohalogen acids) together with chloric (HClO_3_) and iodic (HIO_3_) acids, have been known to be potential carcinogens, experimentally inducing renal tumors [47]. This compound has been classified as possibly carcinogenic to humans (group 2B according to IARC) [48]. Although KIO_3_ has been conferred GRAS status by the FDA [29], it was not listed as a carcinogen with IARC, ACGIH, NTP, or OSHA [49] and did not induce toxic effects under conditions in which the bromate did [29], although similar to KBrO_3_—kidney tissue is presumably more resistant to iodate than other tissues. However, separate studies should be performed to clarify the mechanism of lower kidney sensitivity to iodate.

In the present study we showed that melatonin significantly reduced LPO induced by KIO_3_, when this compound was used at doses corresponding to physiological concentrations of iodine in the thyroid (approx. 9.0 mM) [23,24,25], which is in line with our previous publications [20,21,22]. It should be stressed that the protective effects of melatonin were observed in all examined tissues (when KIO_3_ was applied in concentrations of 10 mM, 7.5 mM and 5 mM), but the most important observation is that melatonin revealed the strongest protective effect in the thyroid gland—it was the only tissue, in which beneficial results of melatonin were observed against as high a KIO_3_ concentration as 15 mM. Additionally, LPO levels resulting from KIO_3_ + melatonin exposure were lower in the thyroid compared to other tissues, but these differences may be due to a weaker damaging effect of KIO_3_ in the thyroid. Further studies are required to clarify the mechanisms responsible for stronger effectiveness of melatonin against KIO_3_-induced damage observed in the thyroid compared to other tissues.

The relationship between melatonin (or its main source, i.e., the pineal gland) and the thyroid gland has been studied for many years. Large experimental evidence suggests the inhibitory action of melatonin on thyroid growth and function [50,51]. These effects were observed when using different experimental models, such as chronic and short-term melatonin administration in vivo, light restriction, pinealectomy or exposure to melatonin under in vitro conditions [50,51]. The inhibitory action of melatonin on the hypothalamic–pituitary–thyroid axis occurs at all three levels, i.e., at the hypothalamic level (inhibition of synthesis and release of thyrotropin releasing hormone (TRH)), at the pituitary level (inhibition of thyrotropin (TSH) release), and directly at the thyroid level, resulting, among other effects, in decreased blood concentrations of thyroid hormones [50,51].

Melatonin is considered as one of the most effective known antioxidants. Mechanisms by which melatonin protects against lipid peroxidation are as follows. Melatonin acts directly by detoxification of reactive oxygen and nitrogen species, like ^•^OH, O_2_^•−^, H_2_O_2_, singlet oxygen (^1^O_2_), HOCl, nitric oxide (NO^•^) or peroxynitrite (ONOO^−^). As an indirect antioxidant, melatonin can stimulate antioxidative enzymes (glutathione peroxidases, glutathione reductase, superoxide dismutase, and catalase) while suppressing the activity of prooxidant enzymes [30]. Furthermore, its metabolites (i.e., AMK—N1-acetyl-5-methoxykynuramine, AFMK—N1-acetyl-N2-formyl-5-methoxykynuramine, and c3OHM—cyclic-3-hydroxymelatonin) can protect against oxidative damage, as similar to melatonin, they are scavengers of hydroxyl- (AMK, AFMK, c3OHM) and hydroperoxyl- (c3OHM) radicals [52,53].

Except for antioxidative properties, melatonin is a regulator of the circadian rhythm and immune system and is also involved in blood pressure and autonomic cardiovascular regulation. Its therapeutic effects have been reported in certain tumors (e.g., breast cancer, ovarian and endometrial carcinoma, prostate cancer, hepatoma and intestinal tumors), cardiovascular diseases or psychiatric disorders [54].

It is worth emphasizing, that short-term use of melatonin, both in animals and humans, is safe, even in extreme doses. Only mild adverse effects (i.e., sleepiness, headache, dizziness or nausea) have been reported [55].

In the current study melatonin was used at a concentration of 5 mM, which, due to its limited solubility, is the highest achievable in vitro concentration and, after all, it is commonly used in experimental studies. This concentration (i.e., 5 mM) is equivalent to ~1.16 × 10^9^ pg/mL. The physiological blood concentration of melatonin in humans is 0–20 pg/mL in the daytime and at night it reaches even 40–200 pg/mL and decreases with age (e.g., [56]). Exogenous melatonin is applied therapeutically in doses between 2 and 10 mg, and the highest dose of melatonin used in clinical trials was 25 mg [57]. The intravenous administration of melatonin at a dose of 25 mg resulted in a blood concentration of ~7.52 × 10^5^ pg/mL [57]. Relating the abovementioned melatonin concentrations to those used by us it should be concluded that the concentrations used in the current experiment exceed by several orders of magnitude the physiological melatonin concentrations and even those resulting from standard doses of exogenous melatonin application; therefore they should be treated as pharmacological.

The melatonin level declines gradually over the life-span, which may cause disorders related to an altered circadian rhythm, such as sleeping disorders, delirium or disorders of cognitive functioning, especially characteristic for the elderly [58]. Moreover, available studies show that disruption of the circadian rhythm or clock gene expression may lead to liver diseases, such as liver steatosis, inflammation or cancer development. These facts may suggest, that supplementation of melatonin not only prevents oxidative stress-induced liver damage (induced e.g., by alcohol drinking or excess fatty acid diet), but also through restoring the circadian rhythm may be a promising therapeutic strategy for liver diseases [59].

In addition, other tissues, examined in the present work, are susceptible to oxidative stress. Especially the brain, with its high oxygen consumption and lipid-rich content can be very prone to this kind of damage [60]. In the ovary, oxygen radicals play important physiological roles, but its cyclic production over years may lead to an increased cumulative risk of ovarian pathology [61]. The small intestine is the main organ involved in the digestion and absorption of nutrients and is directly exposed to drugs and toxic food contaminants [62].

In physiological conditions there is a balance between production of ROS and RNS and their elimination by protective mechanisms, but with aging or under certain conditions, defense mechanisms are not sufficient, which may result in numerous pathologies. For this reason, it is advisable to look for new potential pharmacological agents against known pro-oxidants. In our opinion melatonin—a safe and strong antioxidant—should be considered as a potential protective agent against oxidative damage to membrane lipids caused by KIO_3_ not only in the thyroid gland, but also in other tissues, especially in older people.

Concerning clinical conditions associated with the exposure to KIO_3_ excess, the following should be taken into consideration. Uncontrolled supplementation of tablets or drops containing microgram doses of KIO_3_ seems to be a very probable situation, especially in the older population, while a variety of mineral waters, both with standardized and unstandardized iodine concentration, when drunk in huge amounts may contribute to iodine excess. Iodine contrast agents used in diagnostics and different medications, such as eye drops or antiseptics, commonly used in the general population, contain a very high amount of iodine compounds. Tablets with milligram doses of KIO_3_ used at the time of nuclear emergency, although generally safe, may potentially cause some pro-oxidative effects. Overconsumption of iodized salt does not seem to constitute a strong risk factor of excessive exposure of an individual to iodine, however it should be also taken into account at least at the population level. In such situations of increased exposure to iodine compounds and other external factors with pro-oxidative properties, the potential beneficial effects of antioxidants such as melatonin could be very important. However, it should not be forgotten that our experiment was performed in in vitro conditions; therefore, it may not be directly extrapolated into in vivo situations and, consequently, it may not have a direct impact in clinical practice, at least at the current stage of research.

To our knowledge, our study is the first attempt to compare the protective effects of melatonin against experimentally-induced oxidative damage in various porcine tissues. The differences observed in this work should be confirmed by using other methods and other markers of oxidative damage (not only to membrane lipids but also to DNA and proteins) and, whenever possible, by using additional tissues. We intend to expand our research in this area in the future.

## 5. Conclusions

The thyroid gland is less sensitive to pro-oxidative effects of KIO_3_ when compared to other tissues. Melatonin reveals a strong protective effect against oxidative damage caused by KIO_3_, when this pro-oxidant is used in doses resulting in physiological concentrations of iodine in the thyroid. The strongest protective effect was observed in the thyroid gland which suggests that this organ responds stronger to antioxidative effects of melatonin. However, beneficial effects were significant also in other tissues. Melatonin, as a very safe agent, should be considered to avoid the potential damaging effects of iodine compounds applied in iodine prophylaxis.

## Data Availability

The datasets used and/or analyzed during the current study are available from the corresponding author on reasonable request.
