# Correction: Iwan et al. Pro-Oxidative Effect of KIO_3_ and Protective Effect of Melatonin in the Thyroid—Comparison to Other Tissues. *Life* 2021, *11*, 592

**DOI:** 10.3390/life12071007

**Published:** 2022-07-07

**Authors:** Paulina Iwan, Jan Stepniak, Malgorzata Karbownik-Lewinska

**Affiliations:** 1 Department of Oncological Endocrinology, Medical University of Lodz, 7/9 Zeligowski St., 90-752 Lodz, Poland; paulina.iwan@op.pl (P.I.); jan.stepniak@umed.lodz.pl (J.S.); 2 Polish Mother’s Memorial Hospital—Research Institute, 281/289 Rzgowska St., 93-338 Lodz, Poland

In the original article [1], there was a mistake in Figure 2 as published, namely the graph showing the levels of lipid peroxidation in the ovary was inserted twice and, at the same time, the graph showing the levels of lipid peroxidation in the spleen was not inserted at all. The corrected Figure 2 appears below.

The authors apologize for any inconvenience caused and state that the scientific conclusions are unaffected. The original article has been updated.

## Figures and Tables

**Figure 2:**
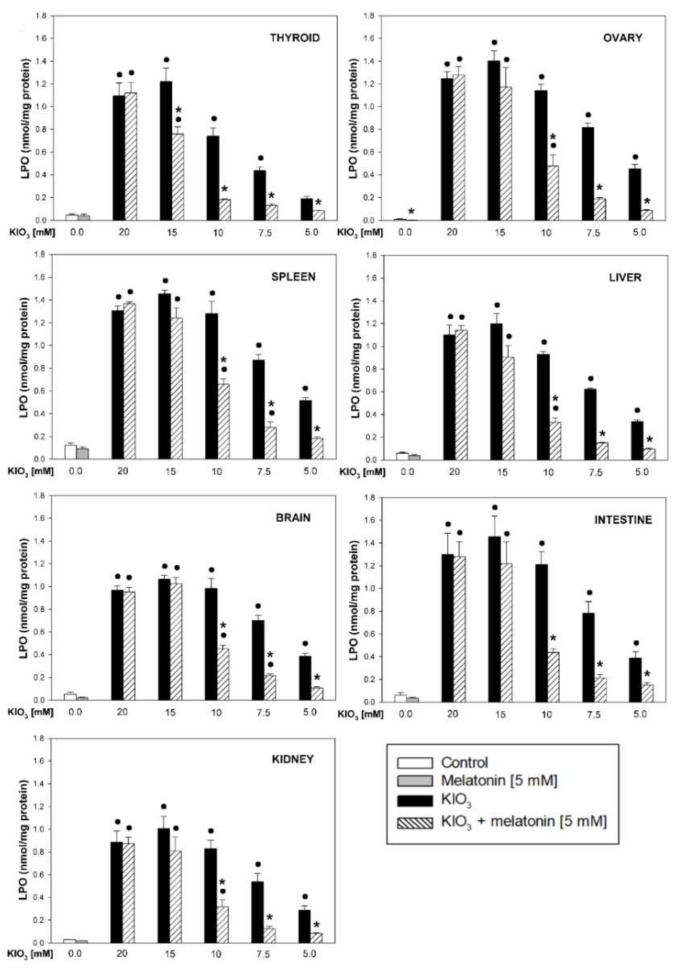
Lipid peroxidation, measured as MDA + 4-HDA level, in homogenates of porcine tissues (thyroid, ovary, spleen, liver, brain, small intestine, kidney), incubated without any substance (control; white bars), or with melatonin (5 mM) (grey bars), or with KIO_3_ (20; 15; 10; 7.5; 5.0 mM) (black bars), or with KIO_3_ (20; 15; 10; 7.5; 5.0 mM) + melatonin (5 mM) (striped bars). ●—*p* < 0.05 vs. respective control (either without any substance or with melatonin); *—*p* < 0.05 vs. KIO_3_ in the same concentration.
